# Two Novel Pathogenic Variants of *TJP2* Gene and the Underlying Molecular Mechanisms in Progressive Familial Intrahepatic Cholestasis Type 4 Patients

**DOI:** 10.3389/fcell.2021.661599

**Published:** 2021-08-24

**Authors:** Jia Tang, Meihua Tan, Yihui Deng, Hui Tang, Haihong Shi, Mingzhen Li, Wei Ma, Jia Li, Hongzheng Dai, Jianli Li, Shengmei Zhou, Xu Li, Fengxiang Wei, Xiaofen Ma, Liangping Luo

**Affiliations:** ^1^NHC Key Laboratory of Male Reproduction and Genetics, Guangdong Provincial Reproductive Science Institute (Guangdong Provincial Fertility Hospital), Guangzhou, China; ^2^Department of Medical Imaging Center, The First Affiliated Hospital of Jinan University, Jinan University, Guangzhou, China; ^3^Medical Genetics Center, Jiangmen Maternity and Child Health Care Hospital, Jiangmen, China; ^4^Department of Molecular and Human Genetics, Baylor College of Medicine, Houston, TX, United States; ^5^College of Life Sciences, University of Chinese Academy of Sciences, Beijing, China; ^6^BGI Genomics Co., Ltd., Shenzhen, China; ^7^Department of Biology, School of Basic Medicine, Jiamusi University, Jiamusi, China; ^8^Department of Pathology and Laboratory Medicine, Children’s Hospital Los Angeles, Los Angeles, CA, United States; ^9^Key Laboratory of Structural Biology of Zhejiang Province, School of Life Sciences, Westlake University, Hangzhou, China; ^10^Longgang District Maternity & Child Healthcare Hospital of Shenzhen City, Shenzhen, China; ^11^Department of Medical Imaging of Guangdong Second Provincial General Hospital, Guangzhou, China

**Keywords:** PFIC, *TJP2*, novel pathogenic variant, cytoskeleton, molecular mechanism

## Abstract

Progressive familial intrahepatic cholestasis (PFIC) is an autosomal recessive inherited disease that accounts for 10%–15% childhood cholestasis and could lead to infant disability or death. There are three well-established types of PFIC (1–3), caused by mutations in the *ATP8B1*, *ABCB11*, and *ABCB4* genes. Biallelic pathogenic variants in the tight junction protein 2 gene (*TJP2*) were newly reported as a cause for PFIC type 4; however, only a limited number of patients and undisputable variants have been reported for *TJP2*, and the underlying mechanism for PFIC 4 remains poorly understood. To explore the diagnostic yield of *TJP2* analysis in suspected PFIC patients negative for the PFIC1–3 mutation, we designed a multiplex polymerase chain reaction-based next-generation sequencing method to analyze *TJP2* gene variants in 267 PFIC patients and identified biallelic rare variants in three patients, including three known pathogenic variants and two novel variants in three patients. By using CRISPR-cas9 technology, we demonstrated that *TJP2* c.1202A > G was pathogenic at least partially by increasing the expression and nuclear localization of TJP2 protein. With the minigene assay, we showed that *TJP2* c.2668-11A > G was a new pathogenic variant by inducing abnormal splicing of *TJP2* gene and translation of prematurely truncated TJP2 protein. Furthermore, knockdown of TJP2 protein by siRNA technology led to inhibition of cell proliferation, induction of apoptosis, dispersed F-actin, and disordered microfilaments in LO2 and HepG2celles. Global gene expression profiling of TJP2 knockdown LO2 cells and HepG2 cells identified the dysregulated genes involved in the regulation of actin cytoskeleton. Microtubule cytoskeleton genes were significantly downregulated in TJP2 knockdown cells. The results of this study demonstrate that TJP2 c.1202A > G and TJP2 c.2668-11A > G are two novel pathogenic variants and the cytoskeleton-related functions and pathways might be potential molecular pathogenesis for PFIC.

## Introduction

Progressive familial intrahepatic cholestasis (PFIC) is a group of liver disorders that are caused by disrupted bile homeostasis. PFIC patients present with intrahepatic cholestasis in infancy or early childhood. The disease accounts for ∼10%–15% of childhood cholestasis and could lead to infant disability or death (Jacquemin, 2012; Srivastava, 2014). Although the exact prevalence of PFIC remains unknown, the estimated incidence ranges between 1 and 50,000–100,000 (Jacquemin, 2012; Srivastava, 2014). Progressive familial intrahepatic cholestasis are broadly divided into six subtypes, according to clinical presentation, laboratory findings, liver histology, and genetic defect. Variants in *ATP8B1*, *ABCB11*, *ABCB4, TJP2, NR1H4*, and *MYO5B* are the main genetic causes for the development of PFIC1, 2, 3, 4, 5, and 6, respectively (Smit et al., 1993; Lam et al., 2010; Paulusma et al., 2010; Sambrotta et al., 2014; Srivastava, 2014; Himes et al., 2020). MYO5B deficiency may lead to isolated cholestasis with normal serum gamma-glutamyl transferase activity (Gonzales et al., 2017). A recent study reported that variants in the *USP53* gene could generate a partial phenocopy of TJP2 disease. *USP53*, is a tight junction (TJ) protein, colocalizes and interacts at the cellular TJs with other tight junction proteins 1 and 2 (TJP1 and TJP2) (Zhang et al., 2020b).

The *TJP2* gene was first discovered and reported by Duclos et al. (1994). It is located at chromosome 9q21.11 and has a total of 140,901 base pairs comprising 23 exons (NM_004817.3). The product of *TJP2* gene is the tight junction protein 2, also called zona occludens 2 (ZO-2), which belongs to the membrane-associated guanylate cyclase family and is involved in the connection between epithelial cells and endothelial cells (Jesaitis and Goodenough, 1994). The TJP2 protein not only binds to the C-terminus of various transmembrane binding proteins but also interacts with multiple nuclear proteins, and participates in the regulation of gene expression and cell proliferation (Gonzalez-Mariscal et al., 2012; Traweger et al., 2013). It is a ubiquitous tight junction scaffold protein present in liver (Jesaitis and Goodenough, 1994). Tight junctions are intercellular barriers that control paracellular solute diffusion across cell layers and separate bile from plasma in liver (McCarthy et al., 2000; Tsukita et al., 2001).

Progressive familial intrahepatic cholestasis type 4 (PFIC4, OMIM #615878) was first described by Sambrotta et al. (2014). The deficiency of TJP2 protein may lead to the disruption of intercellular connections, which facilitates the bile to enter the liver parenchyma through the paracellular space and leads to cholestasis (Sambrotta and Thompson, 2015). Although an increasing number of pathogenic variants have been revealed in the *TJP2* gene (Vitale et al., 2018; Wei et al., 2020; Zhang et al., 2020a), the molecular mechanism by which those variants cause PFIC needed to be further characterized (Sambrotta and Thompson, 2015; Amirneni et al., 2020).

In our studies, we designed a multiplex polymerase chain reaction (PCR)-based next generation sequencing method to analyze *TJP2* gene variants in 267 PFIC patients negative for PFIC 1–3 mutations and identified three known pathogenic variants and two novel variants. Using CRISPR-cas9 and minigene technologies, we confirmed that the two novel variants are likely pathogenic. To better understand the pathogenicity of PFIC 4, we used small interfering RNA (siRNA) technology to downregulate the expression of TJP2 protein and investigated its impact on the cell proliferation, apoptosis, cytoskeleton, and overall structure in LO2 and HepG2 cell lines. Meanwhile, we performed global gene expression profiling of TJP2 knockdown LO2 cells and control LO2 cells to understand the signaling pathways associated with the pathogenesis of TJP2 deficiency. These results are helpful to understand the pathogenesis and may guide the treatment of PFIC.

## Materials and Methods

### Patient Enrollment and Ethical Conduct of Research

Two hundred and sixty-seven PFIC patients with normal γ-glutamyl transpeptidase and free of pathogenic variants in *ABCB11, ATP8B1*, and *ABCB4* were recruited globally by Baylor College of Medicine. We excluded those patients with disease reasonably attributed to drug exposure, autoimmune hepatitis, infection, and biliary atresia. A total of 267 Caucasian patients are sporadic cases and unrelated, including 138 females and 129 males, aged up to 30 years. All patients have signed the written informed consent before participating in this project. This study has been approved by the Institutional Review Board for Human Subject Research at the Jiangmen Maternity and Child Health Care Hospital (Jiangmen, IRB number: 2019053).

### Sample Collection and DNA Extraction

Two milliliters of peripheral blood was collected from each PFIC patient. The genomic DNA was extracted from peripheral blood leukocytes with a commercially available DNA isolation kit (Gentra Systems Inc., Minneapolis, MN), according to the manufacturer’s instructions.

### Multiplex PCR-Based Next Generation Sequencing Analysis of *TJP2* Gene

Twenty-three pairs of *TJP2* gene primers were designed to amplify TJP2 exonic regions (Supplementary Table 1). Multiplex PCR preamplification of the *TJP2* gene was performed using TaqMan^®^ PreAmp Master Mix (Cat no. 4391128, Applied Biosystems) following the manufacturer’s instructions. The PCR products were fragmented and indexed separately for the subsequent sequencing, as described previously (Li et al., 2016), Equal molar ratios of 48 indexed samples were pooled and sequenced on a MiSeq using reagent kit V2 (Illumina, San Diego, CA) with 150-cycle single-end reads. The raw data in basic calling files (.bcl format) were converted to qseq files before demultiplexing with CASAVA software version 1.7 (Illumina). Demultiplexed sequence reads were aligned to the *TJP2* gene reference sequence NM_004817.3, and variants were detected by the NextGENe software version 2.3 (SoftGenetics, State College, PA). Sanger sequencing was used to validate the sequence variants identified by NGS as previously described (Li et al., 2015). Confirmed variants were annotated through a public population database and literature databases including 1000 Genomes (version phase3), dbSNP (build 148), GnomAD^1^, Clinvar (version 201706), HGMD (version 20164), and OMIM^2^. Common variants (frequency > 1% in database) were discarded. Rare or novel variants were further investigated on its amino acid conservation, protein structure, and function. Variants that change located in conserved, structural, or functional regions of protein were regarded as potential pathogenic variants. Variant interpretation was performed according to the American College of Medical Genetics (ACMG) guidelines (Richards et al., 2015).

### Pathogenicity Prediction of Novel Variants and Experimental Validations

For the novel variants, c.1202A > G (p.Glu401Gly) and c.2668-11A > G, their functional impacts were predicted by multiple *in silico* tools software firstly. Then, for missense variant c.1202A > G (p.Glu401Gly), CRISPR-cas9 technology was utilized to construct mutant cell strain (HepG2) to confirm pathogenicity by reverse transcription polymerase chain reaction (RT-PCR), Western blot analysis, cell counting kit-8 assay analysis, and immunofluorescence protein analysis; Minigene assay was utilized to explore the impact of c.2668-11A > G variants on the splicing pattern of TJP2 gene. All experimental procedures and parameters were provided in the supplementary methodology section (Supplementary Methods).

### Functional Validation and Molecular Mechanism Exploration of the Deficiency of TJP2 Protein

Using siRNA technology for downregulated the expression of TJP2 protein in LO2 and HepG2 cell lines separately. For these two TJP2 knockdown cell lines, we analyzed the expression changes of P53 protein and Actin protein by Western blot method, cell proliferation ability by cell counting kit-8 assay, and cell apoptosis rate by flow cytometry. Microtubules and microfilament in LO2 cells were visualized by confocal microscopy using anti-F-actin (1:500; Cytoskeleton, Inc. Cat. # PHDG1-A) and anti-β-tubulin (1:300; Abcam, Cat. #ab195883). Detailed information regarding experimental operations and parameters were provided in the supplementary methodology section (Supplementary Methods).

### RNA-seq Library Preparation, Sequencing, and Analysis

RNA was extracted following the Trizol reagent manual. RNA was precipitated by 1:1 isopropanol (v/v) and 1 μl of glycogen at –20°C overnight. Two parallel mRNA libraries were constructed using VAHTS mRNA-seq V3 Library Prep Kit following the manufacturer’s instructions for each cell line separately. Libraries were sequenced on an Illumina NovaSeq 6000 sequencer for 318 cycles with a strategy of paired end 150. Reads that passed the Illumina quality filters were kept for the subsequent analyses. Adapters were trimmed from the reads, and reads shorter than 17 nt were discarded. The reads were mapped to the human mRNA reference database using FANSe3 algorithm on Chi-Cloud NGS Analysis Platform (Chi-Biotech Co. Ltd., Shenzhen, China). Differentially expressed genes (DEGs) were determined by R package edgeR (Version: 3.28.1) at the following cutoffs: *p*-value < 0.01 and absolute log2 fold change > 1 (Robinson et al., 2009; Rajkumar et al., 2015). Kyoto Encyclopedia of Genes and Genomes (KEGG) signaling pathway enrichment analyses were performed for DEGs by clusterProfiler (Version: 3.14.3) (Alexa et al., 2006; Yu et al., 2012). Kyoto Encyclopedia of Genes and Genomes pathways with the Benjamini-Hochberg-adjusted *p*-value < 0.05 were considered statistically significant.

### Quantitative Real-Time PCR

Total RNA was isolated with TRIzol reagent according to the manufacturer’s instructions. Three parallel RNA samples were reverse transcribed using random hexamer primers in the presence of RNase inhibitor (Takara Bio, Shiga, Japan). qRT-PCR was performed with SYBR Premex EX Taq (Takara Bio) using the 7300 Sequence Detection System (Applied Biosystems, Foster City, CA). A relative quantification analysis was performed using the ΔΔCt method (Livak and Schmittgen, 2001), with actin as endogenous references. Relative gene expression is presented as the ratio of the target gene to reference.

### Statistical Analysis

Statistical analyses were performed with SPSS v. 19.0. The data were performed in triplicate and analyzed by using mean ± SD, Student’s unpaired *t*-test. The value of *n* is mentioned in the figure legends and always stands for separate biological replicates. All comparisons between groups were made by unpaired two-tailed Student’s *t*-test. Differences with ^∗^ *p* < 0.05, ^∗∗^*p* < 0.01, and ^∗∗∗^*p* < 0.001 were considered statistically significant. n.s., not significant.

## Results

### Variants Detected in Patients by Multiplex PCR-Based Sequencing of *TJP2* Gene and Clinical Features

Five *TJP2* gene variants were detected in the 267 PFIC patients, including two novel variants and three known pathogenic variants (Table 1). The two novel variants are c.1202A > G (p.Glu401Gly) and c.2668-11A > G, which were confirmed by Sanger sequencing (Supplementary Figure 1). Two compound heterozygous variants were detected in patient 46384 and patient 54691. The homozygous variants were identified in patient 62966. Table 1 summarizes clinical features and ACMG classification information.

**TABLE 1:** Five types of *TJP2* gene variants detected in three patients.

**ID**	**Gender**	**Date of birth**	**Age of onset**	**Clinical indication**	**GGT (U/L)**	**Variant 1 (NM_004817.3, NP_004808.2)**	**rsID**	**ClinVar**	**ACMG classification (evidence levels)**	**Allele frequency**	**Histologic features of cholestasis**	**Survival status**
46348	F	4/14/1994	NA	Neonatal cholestasis, liver failure	Normal	c.817delG, p.Ala273Profs*38	rs864321697	Pathogenic	Pathogenic (PVS1, PM2, PP3, PP4, PP5)	NA	NA	Alive
						**c.1202A > G,p.Glu401Gly**	rs1057515614	NA	Likely Pathogenic (PS3, PM2, PP3, PP4)	G = 0.000004, 1/251308 (GnomAD_exomes)		
54691	F	6/30/2010	Neonatal onset	Intermittent jaundice of neonatal onset, cirrhotic liver, presented with liver failure.	49 (5–55), Normal	c.2438dup, p.Asn814Glnfs*28	rs776869985	Pathogenic	Pathogenic (PVS1, PM2, PP3, PP4, PP5)	NA	Hepatocytes with prominent rosetting had bile-tinged cytoplasm and scant giant-cell transformation. Canalicular lumina often contained bile pigment, as did the cytoplasm of Kupffer cells. Portal-tract cholestasis and ductular reaction were not seen.	Alive
						c.2668-1G > GT	rs864321695	Pathogenic	Pathogenic (PVS1, PM2, PP3, PP4, PP5)	NA		
62966	F	6/13/2009	26 months	Liver carcinoma, cholestatic jaundice, hepatosplenomegaly, hepatic dysfunction	Normal	**c.2668-11A > G(Hom)**	rs933238834	NA	Likely Pathogenic (PS3, PM2, PP3, PP4)	G = 0.000009,2/232746 (GnomAD_exomes)	The liver parenchyma shows patchy cellular swelling of hepatocytes as well as patchy hepatocellular and canalicular cholestasis. The portal areas show piecemeal necrosis as well as ductular proliferation. Additionally, there is moderate lobular mixed inflammatory infiltrate. Microvesicular and macrovesicular steatosis is present in about 20% of the tissue. Extensive bridging fibrosis and regenerative nodules are evident by trichrome special stain.	Alive

*Survival status: follow-up till 2015.*

*Bold value means novel variant.*

Biochemical data of patient 54691 at 26 months are serum alpha-fetoprotein level (171,000 ng/ml, ≤ 13.4), ALP (145 U/L, 80–220), AST (192 U/L, 15–46), ALT (114 U/L, 3–15), total bilirubin (10.2 mg/dl, 0.1–1.3), triglycerides (64 mg/dl, 35–135), albumin (2.7 g/dl, 3.8–5.4), and platelets (128 K/μl, 150–450). We perform histologic and immunohistochemical studies (anti-TJP2 and anti-CLDN1 immunostaining) on patient 54691 (Zhou et al., 2015).

Patient 62,966 at 5 years old has a weight of 17.50 kg, a height of 105.50 cm, a BMI of 15.72, a weight percentile of 15, a height percentile of 3, and a BMI percentile of 63, has cholestatic jaundice (idiopathic) and hepatitis, fat-soluble vitamin deficiency of ADEK, splenomegaly, hypervascular lesions in liver, elevated AFP (most recently normal), and scleral icterus. Seven-year-old patient 62,966 has idiopathic cholestatic neonatal hepatitis with striking hepatomegaly and splenomegaly. She has a prolonged INR, which may be related to vitamin K deficiency or worsening synthetic function, despite vitamin K replacement. Her albumin is also low, raise concern for worsening synthetic function. Her absorption of all fat-soluble vitamins is very poor despite high intake, as seen in disorders where there is poor bile acid delivery to the gut. Her declining platelet count and increasing spleen size indicated hypersplenism due to portal hypertension. CT examination found three masses. In the anterolateral dome of the liver, corresponding to the junction between Couinaud segment 8 and segment 4A, there is a hypervascular liver mass measuring 2.5 × 2.4 × 2.1 cm. During portal venous phase imaging, the mass becomes iso-attenuating to liver parenchyma. During delayed phase imaging, there is subtle central lower attenuation with a rim of continued slightly higher attenuation relative to surrounding liver parenchyma. We also conducted hematoxylin-eosin staining assays on patient 62966 (Table 1).

### The *TJP2* c.1202A > G Is a Novel Pathogenic Variant in PIFC

**FIGURE 1:**
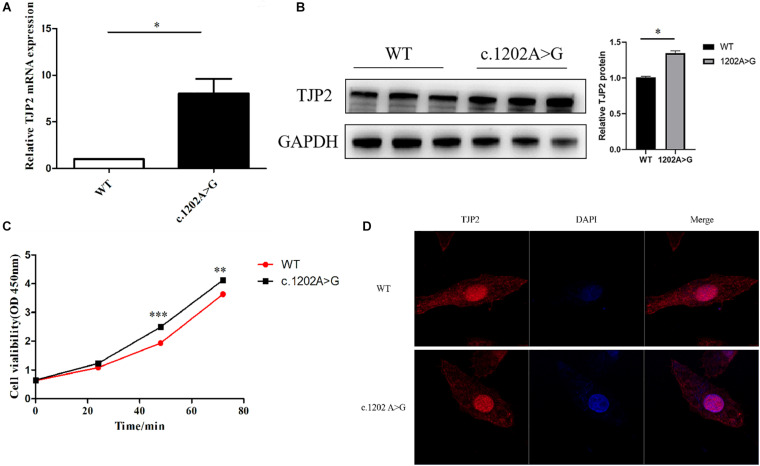
The *TJP2* c.1202A > G (p.Glu401Gly) is a novel pathogenic variant. **(A)** The mutant HepG2 cells show a significantly higher *TJP2* mRNA expression level than the wild-type HepG2 cells. **(B)** The mutant HepG2 cells show a significantly higher TPJ2 protein expression level than the wild-type HepG2 cells. **(C)** The cell viability was significantly enhanced in mutant HepG2 cells at 48 h as compared to wild-type HepG2 cells. **(D)** TJP2 protein was enriched in the nucleus of TJP2 c.1202A > G mutated HepG2 cells. DAPI indicates that the nuclei are stained with 4’,6-diamidino-2-phenylindole, dihydrochloride (DAPI). Merge represents the merged fluorescent image of nucleus and cytoplasm labeled by red and blue fluorescence.

The missense variant TJP2 c.1202A > G (p.Glu401Gly) was predicted as probably damaging (HDIV score: 0.95, HVAR score: 0.714) by PolyPhen-2,Disease_causing (1.000) by MutationTaster, deleterious (score: –4.05) by PROVEAN, and damaging (score: 0.013) by SIFT. Based on the GERP++, PhyloP, and PhastCons methods, this missense variant position probably belongs to a conserved element as the conservation scores are 5.75, 4.794, and 1 separately. We used CRISPR-cas9 technology to construct a mutant HepG2 cell line and performed various experiments to further characterize the functional impact of the variant. The expression levels of TJP2 mRNA and protein were upregulated (Figures 1A,B). Cell proliferation was enhanced in c.1202A > G mutated HepG2 cells as compared to wild-type HepG2 cells at 48 h after transfection (*p*-value < 0.05, Student’s *t*-test, Figure 1C). The immunofluorescence assay demonstrated that *TJP2* protein expressed at both cytoplasm and nucleus in WT HepG2 cells. Almost the *TJP2* protein is expressed at nucleus in c.1202A > G mutated HepG2 cells (Figure 1D). These results suggest that *TJP2* c.1202A > G is pathogenic at least partially by increasing the expression and nuclear localization of TJP2 protein. According to the ACMG guidelines, our prediction results and functional experimental discoveries above could supply PS3, PM2 (only one heterozygote in gnomAD, 4.0E–06), PP3, and PP4 evidence for the *TJP2* c.1202A > G variant. Combining with all these evidence categories, we suggest that TJP2 c.1202A > G is a likely pathogenic variant.

### The *TJP2* c.2668-11A > G Variant Leads to Translation of Prematurely Truncated TJP2 Protein

**FIGURE 2:**
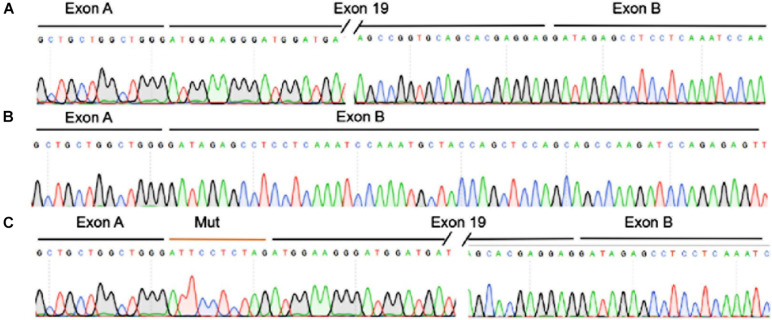
The *TJP2* c.2668-11A > G is a new pathogenic variant by inducing abnormal splicing of *TJP2* gene. **(A)** The transcription of TJP2 exon 19 of the wild-type plasmid was validated by minigene assay and sanger sequencing. **(B)** The transcript of the empty plasmid. **(C)** The transcription of exon 19 and retained 10 bp intronic region (ATTCCTCTAG) of the mutant plasmid as validated by minigene assay and Sanger sequencing.

The *TJP2* c.2668-11A > G variant is located in the intron region of *TJP2* gene and may have an impact on splicing. We utilized Alamut Visual mutation analysis software (version 2.14, Interactive Biosoftware, Rouen, France), including five different algorithms, SpliceSiteFinder-like, MaxEntScan, NNSPLICE, GeneSplicer, and Human Splicing Finder, to evaluate the splicing effects of the variant. Alamut Visual mutation analysis software predicted that the *TJP2* c.2668-11A > G variant created a new acceptor site and a 10-bp intron region was retained in the transcript, causing premature translational termination of TJP2 protein (Supplementary Figure 2). The conservation scores predicted by the PhyloP and PhastCons methods are 0.669 and 0.903, indicating that the variant position might be a conserved element. Then, we performed a minigene experiment to further validate the prediction. The results of the minigene assay showed the plasmid containing wild-type TJP2 could transcribe exon 19 of the TJP2 gene. In addition to exon 19, the plasmid containing mutant TJP2 expressed an additional 10-bp intronic region (ATTCCTCTAG) (Figure 2, Supplementary Figure 3). These results suggest that the *TJP2* c.2668-11A > G variant causes abnormal splicing of TJP2 mRNA; translation of 897 amino acids prematurely truncated TJP2 protein (normal is 1190 amino acids, Supplementary Table 4). According to the ACMG guidelines, our prediction results and functional experimental discoveries above could supply PS3, PM2 (only two heterozygotes in gnomAD, 9.0E–06), PP3, and PP4 evidence for the TJP2 c.2668-11A > G variant. Combining with all these evidence categories, we suggest that TJP2 c.2668-11A > G is a likely pathogenic variant.

### Knockdown of TJP2 Protein Inhibits Cell Proliferation and Induces Apoptosis

**FIGURE 3:**
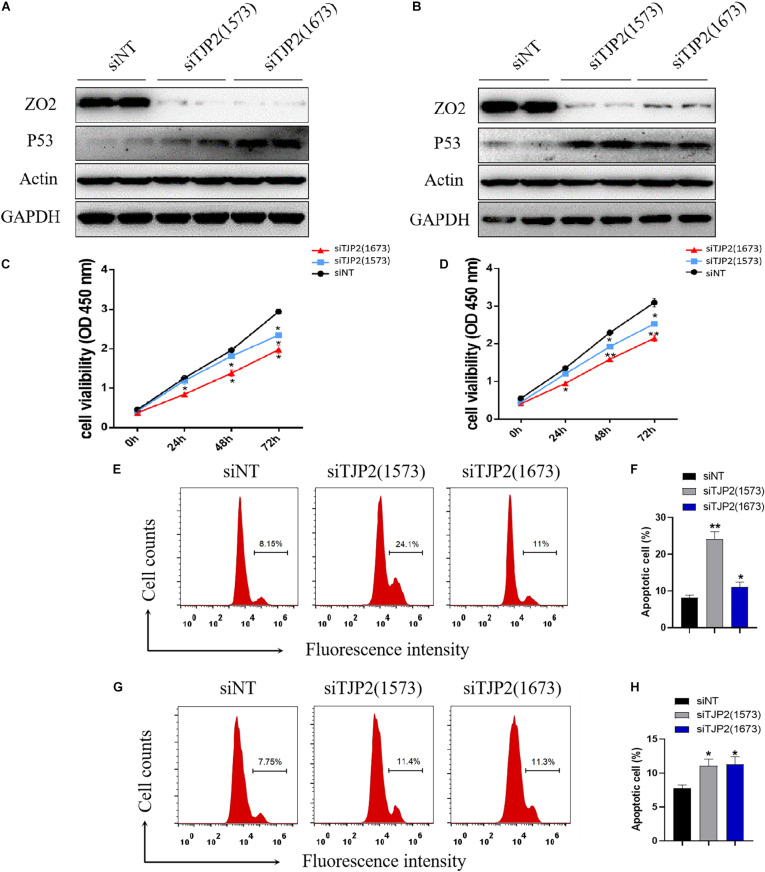
TJP2 protein deficiency leads to an increase in TP53 protein expression, inhibition of cell proliferation and induction of apoptosis in LO2 and HepG2 cells. **(A)** Downregulation of the *TJP2* gene by two siRNAs (1573 and 1673) remarkably decreased the TJP2 protein expression and increased TP53 protein expression in LO2 cells in comparison with negative control (siNT). **(B)** Downregulation of TJP2 protein by two siRNAs (1573 and 1673) dramatically decreased the TJP2 protein expression and increased TP53 protein expression in HepG2 cells in comparison with negative controls. **(C)** Knockdown of TJP2 protein by siRNAs caused a significant decrease in cell proliferation in LO2 cells at 48 h and 72 h after transfection of siRNAs. **(D)** Knockdown of TJP2 protein by siRNAs caused a significant decrease in cell proliferation in HepG2 cells at 48 h and 72 h after transfection of siRNAs. **(E,F)** Silencing TJP2 protein expression by siRNAs induced cell apoptosis in LO2 cells. **(G,H)** Silencing TJP2 protein expression by siRNAs induced cell apoptosis in HepG2 cells. The data is presented as mean ± standard deviation (*n* = 3, *p < 0.05, **p < 0.01). ZO2, 150 kDa; P53, 53 kDa; ACTIN, 42 kDa; GAPDH, 37 kDa.

To investigate the molecular mechanism underlying the pathogenesis of *TJP2* deficiency, we performed knockdown of TJP2 gene expression by two different siRNAs (1573 and 1673). As compared to the negative control, the siRNAs caused marked reduction in TJP2 protein expression in both LO2 and HepG2 cell lines. As a result, the knockdown of TJP2 protein led to increased expression of TP53 protein in the two cell lines (Figures 3A,B). The CCK-8 assay and flow cytometry assay demonstrated that the downregulation of TJP2 protein expression inhibited cell proliferation at 48 h and 72 h after transfection of siRNAs (Figures 3C,D) and promoted cell apoptosis in both LO2 and HepG2 cells (*p* < 0.05 for all cases, Student’s *t*-test, Figures 3E–H).

### Knockdown of TJP2 Causes Cell Structural Disorder

**FIGURE 4:**
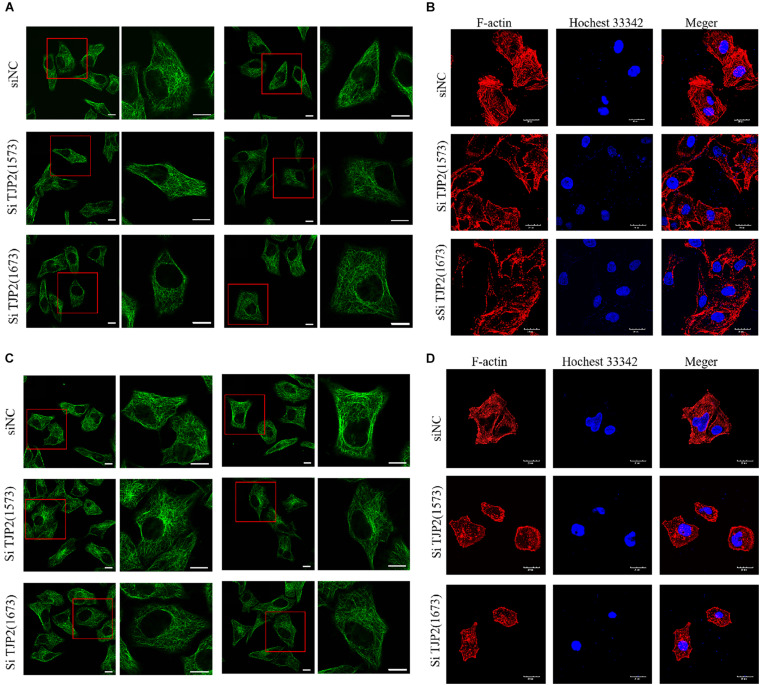
The downregulation of TJP2 expression results in dispersed F-actin and disordered microfilaments. **(A)** No apparent change of microtubules was observed between the negative control group and siRNA-transfected groups in LO2 cells. siNT is the negative control group; siTJP2 (1573) and siTJP2 (1673) are treatment groups transfected with siRNA (1573) and siRNA (1673), respectively. The red box indicates microtubules labeled with green fluorescence. Microtubules in LO2 cells were visualized by confocal microscopy using an anti-β-tubulin antibody (Abcam, Cat. #ab195883). **(B)** The downregulation of TJP2 protein expression resulted in dispersed F-actin and disordered microfilaments in LO2 cells. F-actin (Cytoskeleton Inc., Cat. # PHDG1-A) was labeled with red fluorescence; the nucleus was labeled with blue fluorescence by hochest33342 staining. Merge represents the merged fluorescent image of F-actin and nucleus labeled by red and blue fluorescence. **(C)** No apparent change of microtubules was observed between the negative control group and siRNA-transfected groups in HepG2 cells. **(D)** The downregulation of TJP2 protein expression resulted in dispersed F-actin and disordered microfilaments in HepG2 cells. Scale bars, 20 μm.

To analyze the impact of deficiency of TJP2 protein on the cytoskeleton and overall structure, the cytoskeleton was stained by immunofluorescence with β-tubulin and F-actin antibody, respectively. The cytoskeletons of LO2 and HepG2 cells were observed by confocal laser microscopy. The results showed that silencing TJP2 protein expression did not significantly change the tubulin of LO2 and HepG2 cells. In contrast, the downregulation of TJP2 protein expression resulted in dispersed F-actin and disordered microfilaments (Figure 4), indicating the role of TJP2 in F-actin regulation.

### Functional Characterization of DEGs in TJP2 Knockdown LO2 and HepG2 Cells

**FIGURE 5:**
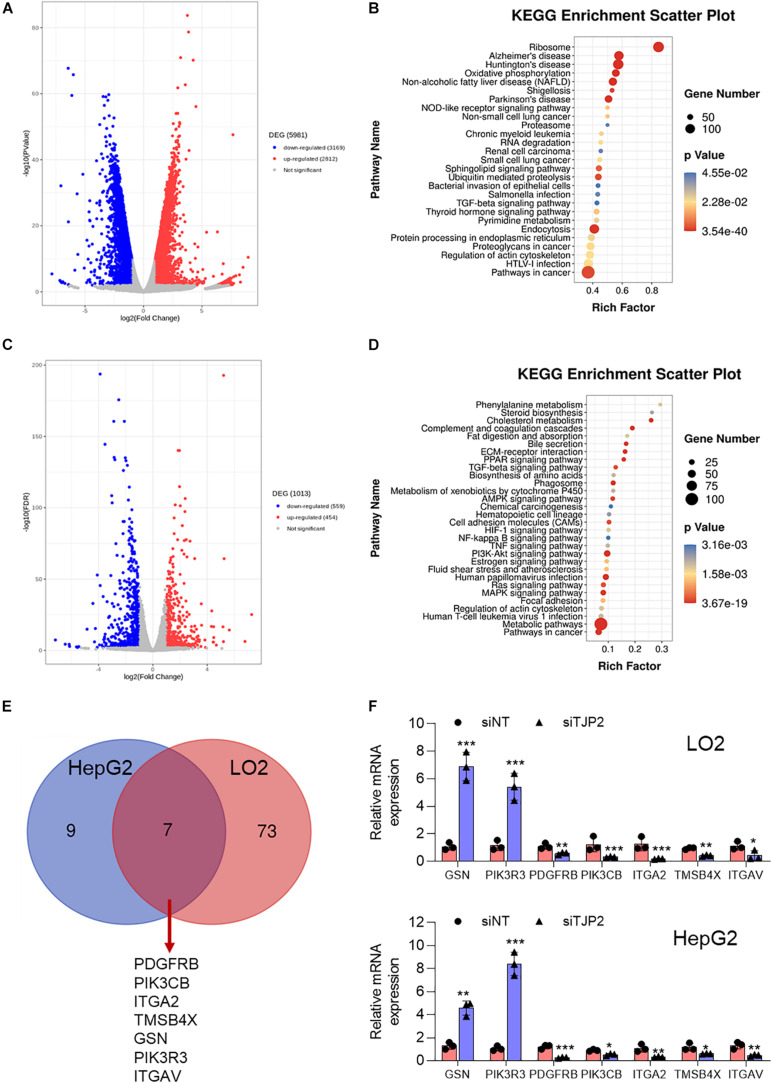
Characterization of DEGs in TJP2 knockdown LO2 and HeG2 cells. **(A)** Volcano plot of the DEGs in TJP2 knockdown LO2 cells, with red and blue dots indicating upregulated and downregulated genes, respectively. **(B)** KEGG signaling pathway enrichment of abnormally expressed genes for TJP2 knockdown LO2 cells. **(C)** Volcano plot of the DEGs in TJP2 knockdown HepG2 cells, with red and blue dots indicating upregulated and downregulated genes, respectively. **(D)** KEGG signaling pathway enrichment of abnormally expressed genes for TJP2 knockdown HepG2 cells. **(E)** Venn diagram showing overlap between the dysregulated genes related to “Regulation of Actin cytoskeleton” in TJP2 knockdown LO2 cells and HepG2 cells. The overlapping genes are indicated below. **(F)** Quantitative PCR confirmation of dysregulated genes. The relative mRNA expression data are presented as mean ± standard deviation (*n* = 3, *p < 0.05, **p < 0.01, ***p < 0.001). siTJP2, siTJP2 (1673).

To understand the signaling pathways associated with pathogenic variants in PIFC, we next performed global gene expression profiling on TJP2 knockdown LO2 cells and HepG2 cells as compared to control cells. In LO2 cells, a total of 5981 DEGs were detected (*p* < 0.01), of which 3,169 were downregulated and 2,812 were upregulated in TJP2 knockdown cells (Figure 5A). In HepG2 cells, a total of 1,013 DEGs were detected (*p* < 0.01), of which 559 were downregulated and 454 were upregulated in TJP2 knockdown cells (Figure 5C). We performed KEGG pathway analysis to find out the overlap pathway of these two cell lines. The overlap KEGG pathways that are significantly enriched for DEGs were pathways in cancer, regulation of actin cytoskeleton, and TGFβ signaling pathway. Related to the functional observation of TJP2 above, the regulation of actin cytoskeleton pathway stood out in the analysis (Figures 5B,D). We compared the LO2 transcriptome data with the data of HepG2 cells. The abnormally expressed genes involved in the regulation of the actin cytoskeleton pathway showed a significant overlap (Figure 5E); these genes are PDGFRB, PIK3CB, ITGA2, TMSB4X, GSN, PIK3R3, and ITGAV. By performing qPCR analysis, we further confirmed the dysregulation of these genes in LO2 and HepG2 cells upon TJP2 knockdown (Figure 5F).

## Discussion

In the present study, we identified three patients among 267 PFIC patients which were previously tested negative for *ABCB11*, *ATP8B1*, and *ABCB4* gene variants carried homozygous or compound heterozygous disease-causing variant in *TJP2* gene. The remaining negative 264 PFIC patients probably have variants outside these areas like the untranslated regions or the other genes (e.g. *NR1H4*, *MYO5B*, *USP53*), especially in patients who experienced cholestatic symptoms within the first year of life (Dröge et al., 2017). Two variants among all the five variants we have identified in *TJP2* gene could be pathogenic based on our functional studies. *TJP2* c.1202A > G was predicted as a damaging mutation by multiple software and could significantly upregulate the expression of TJP2 protein especially in the nucleus, which might lead to abnormal biological processes including enhancement of cell proliferation. Functional prediction and experimental results both indicated that *TJP2* c.1202A > G is most likely a pathogenic variant. Splicing variant *TJP2* c.2668-11A > G causes abnormal splicing of *TJP2* mRNA, which could produce prematurely truncated TJP2 protein and cause the deficiency of TJP2 protein. Deficiency of TJP2 protein has been reported as the pathogenic mechanism of PFIC type 4 (Zhang and Yu, 2016).

Generally, PFIC type 4 predominantly occurs in childhood (Vitale et al., 2018), but patient 46384 who has compound heterozygous pathogenic variants (c.1202A > G and c.817delG, which were inherited from her mother and father, respectively.) was 21 years old at the time of the sample collection. The compound heterozygous variants are a novel missense variant c.1202A > G (p.Glu401Gly) and a pathogenic variant c.817delG (p.Ala273Profs^∗^38) (Sambrotta et al., 2014; Zhou et al., 2015). *TJP2* c.1202A > G could upregulate the expression of TJP2 protein and TJP2 c.817delG caused the deficiency of TJP2 protein. We speculate that the two functionally opposite heterozygous pathogenic variants might lead to a functional balance and partially explain why patient 46384 could survive so long.

Hepatocytes exhibit a unique cellular polarity and the maintenance of cellular polarity is essential for many functions of hepatocytes (Gissen and Arias, 2015). In a recent study, Itoh et al. inactivated the *TJP2/ZO-2* gene in alive mouse livers and observed that the cellular polarity was compromised (Itoh et al., 2021). The specific transporters localized at basolateral and apical membrane domains are responsible for the uptake and secretion processes (Stapelbroek et al., 2010). A study from Sambrotta et al. shows that deficiency of TJP2 protein caused a failure of localization of claudin-1 at the canalicular membrane, with the disruption of the tight-junction structure leading to a leakage of the biliary components through the paracellular space into the liver parenchyma, causing damage to the surrounding hepatocytes and cholangiocytes (Sambrotta and Thompson, 2015). However, the effect of TJP2 protein deficiency on the cytoskeleton and overall cell structure has not been studied. We have used siRNA to study and found that the deficiency of the TJP2 protein could cause filament arrangement disorder and cell fusion in both HepG2 and LO2 cell lines. The mRNA-seq analysis also showed that the dysregulation genes are related to cytoskeleton organization or cytoskeletal binding function.

TJP2 protein often interacts with multiple nuclear proteins to regulate gene expression and cell proliferation (Gonzalez-Mariscal et al., 2012). According to Western blot analysis, we found that the deficiency of TJP2 protein could increase the expression of P53 protein. However, the increased expression of TP53 has turned out not to be statistically significant in the DEG analysis. Knockdown of TJP2 inhibits cell proliferation, induces apoptosis, and causes cell structural disorder. TJP2 is a multidomain molecule that binds to a variety of cell signaling proteins, to the actin cytoskeleton, and to gap, tight, and adherens junction proteins, and inhibits the Wnt signaling pathway, reduces cell proliferation, and promotes apoptosis (Gonzalez-Mariscal et al., 2012). Actin sedimentation studies showed TJP2 protein to interact directly with F-actin (Wittchen et al., 1999). We report that the downregulation of TJP2 protein expression resulted in dispersed F-actin and disordered microfilaments. Based on the results of mRNA-seq analysis in LO2 cells, we found that the deficiency of TJP2 protein could influence the expression of genes involved in intracellular membrane-related organelles and cytoskeleton, thereby affecting the binding function, metabolic processes, and cytoskeleton organization. These biological processes are closely related to the physiological processes related to cholestasis (Javitt, 1994; Chiang, 2009; Boyer, 2013). The KEGG pathway enrichment analysis showed that many DEGs were involved in the apoptosis pathway, which, to a large extent, explains why TJP2 deficiency increases cellular apoptosis.

Reported patients with TJP2 deficiency display severe progressive cholestatic liver disease in early childhood, which increases the risk of developing hepatocellular carcinoma (Zhou et al., 2015). Many studies have reported that infant liver cancer patients have *TJP2* gene variants (Zhou et al., 2015; Parsons et al., 2016; Zhang and Yu, 2016). In our study, the signal pathways enriched for DEGs are mostly related to the small-molecule metabolic pathways and cancer-related signal pathways such as the mTOR signaling pathway, hepatocellular carcinoma, TNF signaling pathway, and mTOR signaling pathway. The dysregulated cancer-associated pathways caused by the deficiency of TJP2 protein might be attributable to the development of hepatocellular carcinoma. Therefore, the influence on cytoskeleton organization pathway and the filament arrangement disorder might provide new insight into the pathogenic mechanism of variants in *TJP2* gene in PFIC type 4.

## Conclusion

This study showed the diagnostic yield of TJP2 analysis in suspected PFIC parents negative for identifiable PFIC1–3 mutations (up to 1.12%). We reported two novel pathogenic variants, TJP2 c.1202A > G and TJP2 c.2668-11A > G, and expanded the clinical and molecular spectrum of PFIC4. Our study enhanced understanding of the consequences of TJP2 deficiency *in vitro* and revealed that the cytoskeleton-related functions and pathways might be potential molecular pathogenesis for PFIC.

## Data Availability Statement

The sequencing data has been deposited in Sequence Read Archive (SRA) with BioProject ID PRJNA702616. All data are released and can be accessed from this BioProject in the submission portal https://www.ncbi.nlm.nih.gov/Traces/study/?acc=PRJNA702616.

## Ethics Statement

This study has been approved by the Institutional Review Board for Human Subject Research at the Jiangmen Maternity and Child Health Care Hospital (Jiangmen, IRB number: 2019053). Written informed consent to participate in this study was provided by the participants’ legal guardian/next of kin.

## Author Contributions

JT, YD, and HT designed the study and revised the manuscript. JT and MT drafted the manuscript and interpreted the data. LL, JLL, FW, XM, ML, and JL reviewed and finalized the manuscript. WM, HD, SZ, HS, and XL conducted the study and collected the data. All the authors have read and approved the final version of the submitted manuscript.

## Conflict of Interest

MT and JL were employed by the company BGI Genomics Co., Ltd. The remaining authors declare that the research was conducted in the absence of any commercial or financial relationships that could be construed as a potential conflict of interest.

## Publisher’s Note

All claims expressed in this article are solely those of the authors and do not necessarily represent those of their affiliated organizations, or those of the publisher, the editors and the reviewers. Any product that may be evaluated in this article, or claim that may be made by its manufacturer, is not guaranteed or endorsed by the publisher.

## Footnotes

1
http://gnomad-old.broadinstitute.org

2
http://www.omim.org
