# Supplementary figures and images for: Two Novel Pathogenic Variants of TJP2 Gene and the Underlying Molecular Mechanisms in Progressive Familial Intrahepatic Cholestasis Type 4 Patients
Source: Front Cell Dev Biol. 2021 Aug 24;9:661599. doi: 10.3389/fcell.2021.661599 (PMC8421653; doi:10.3389/fcell.2021.661599)

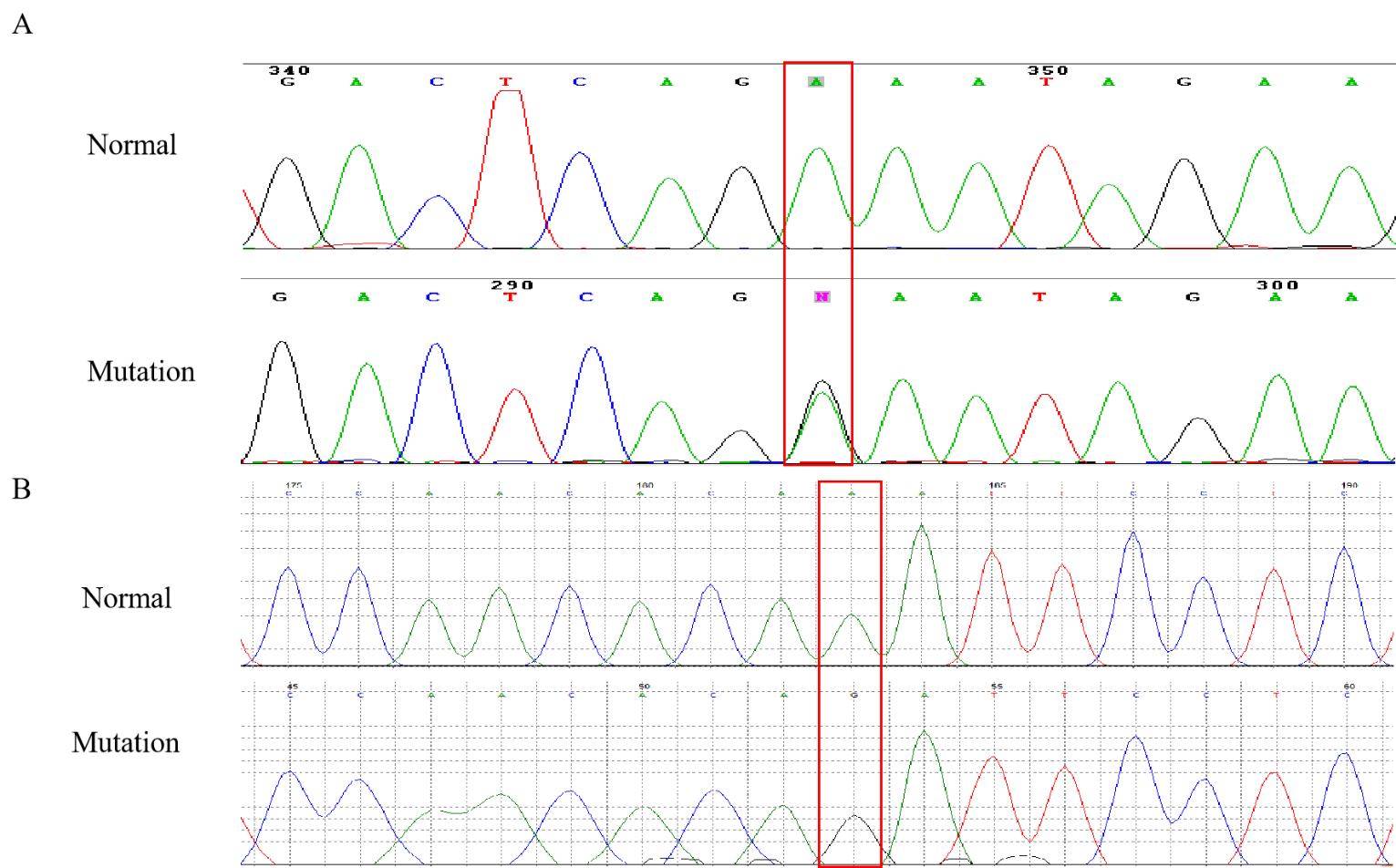

Supplement: Supplementary file 1 [file Image_1.tif]

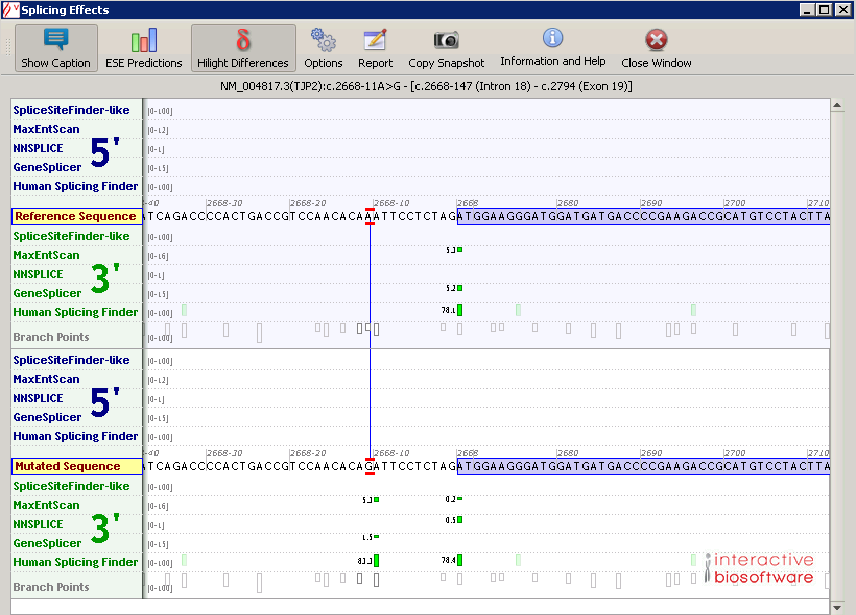

Supplement: Supplementary file 2 [file Image_2.tif]

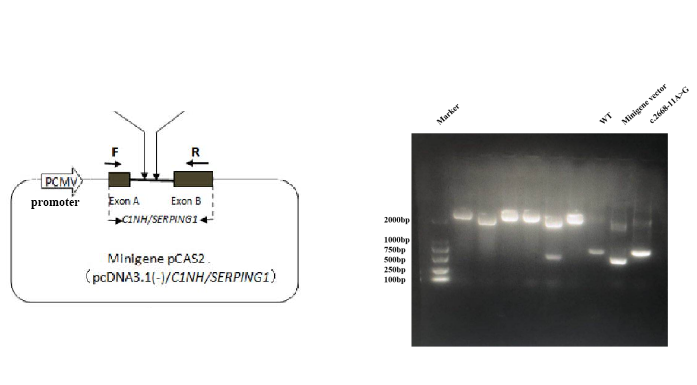

Supplement: Supplementary file 3 [file Image_3.tif]
