# Supplementary material for: Two Novel Pathogenic Variants of TJP2 Gene and the Underlying Molecular Mechanisms in Progressive Familial Intrahepatic Cholestasis Type 4 Patients
Source: Front Cell Dev Biol. 2021 Aug 24;9:661599. doi: 10.3389/fcell.2021.661599 (PMC8421653; doi:10.3389/fcell.2021.661599)
Supplement: Supplementary file 4 [file Data_Sheet_1.doc]

# PART ONE. Supplementary Methodology Section

**CRISPR-cas9 technology**

Plasmid vectors including lentiCRISPRv2 and ZV246 were used for TJP2 c.1202A>G knock in HepG2 cell by CRISPR-Cas9. The lentiCRISPRv2 sgRNAs sequences was as; 5′-caccgGACAGTGACTCAGAAATAGA-3′. TJP2 c.1202A>G single-guide RNA (sgRNA) was synthesized and cloned into lentiCRISPRv2. The ZV246 plasmid is the homologous arm of TJP2 c.1202A>G. Briefly, HepG2 cells were seeded into a 6-well plate. After 24 h that the cells reached 60–70% confluent, the culture media were removed and the cells were washed with phosphate buffer saline (PBS), Plasmid with Lipofectamine 3000 transfection reagent was added to the well. After 48h add 1.5 mL selection medium for monoclonal cell selection (complete medium containing 2 μg/mL puromycin). The cell line construction with knock in of TJP2 c.1202A>G was verified by genomic sequencing.

# Minigene molecular cloning, transfection, and RT-PCR

Mutagenesis was carried out according to the PCR mutagenesis protocol Site directed mutagenesis. Wild-type (wt) minigene *TJP2* exon 19 with intronic was used as a template to generate variants. After construction *TJP2* gene variants, we subjected exon 19 of TJP2 with the intronic boundaries to PCR-Sanger sequencing. PCR was performed under the following conditions: initial denaturation at 95°C for 3 min, followed by 35 cycles of 95 °C for 30 s, 60 °C for 30 s and 72 °C for 30 s.

The identified intronic variant of c.2668-11A>G is located in the neighboring region of the splicing acceptor site of intron 18. To study the effect of this variant on the splicing pattern, a DNA fragment of 477 bp encompassing exon 19 and the flanking intronic sequences of TJP2 was amplified from the genomic DNA.Each PCR product was digested with the BglII and MluI restriction enzymes and cloned into the pCAS2 vector(pcDNA3.1(-)/C1NH/SERPING1), which had also been digested with BamHI and MluI.All of the selected clones were sequenced, and the verified clones(primers,1F CATTGTACCAAAGCCAAGTGATCT,1R GAATGCCTTCCACAGCACCCT), referred to as the wild-type (pTJP2-c.2668-11A) and mutant (pTJP2-c.2668-11G) clones,were retained for expression experiments.

# Minigene splicing assay

The 293T cells were grown in a 5% CO2 incubator at 37 °C in Dulbecco’s modified Eagle’s medium supplemented with 10% fetal bovine serum (Bovogen). 293T cells that had been seeded at 70% confluence in 60 mm dishes 24h before transfection were transfected with 5 μg of pTJP2-c.2668-11A,pTJP2-c.2668-11G and the empty pCAS2 vector using Lipofectamine 3000(from Invitrogen).Twenty-four hours after transfection, cells were collected and total RNA was extracted using TRIzol (Invitrogen).Then, 1.5 μg of total RNA was reverse transcribed using a Reverse Transcription System according to the manufacturer’s instructions(Invitrogen). Following RNA retrotranscription, 400 ng of complementary DNA from the three constructs mentioned above was PCR amplified using the primers (pCAS2-RT-F1 CTGACCCTGCTGACCCTCCT andpCAS2-RT-R1 TTGCTGAGAAGGCGTGGTAGAG).The PCR products were then separated on a 2% agarose gel, and individual bands were excised and sequenced using.

**Cell lines and cell culture**

LO2 and HepG2 cells were obtained from ATCC. Cells were cultured in RPMI 1640 containing 10% FBS, 100 units/ml penicillin, and 100 ug/ml streptomycin at 37 °C in a humidified incubator with 5% CO_2_.

**RNA interference**

One day before transfection, cells were seeded on 6-well plates at 30-50% confluency and then transfected with 50 nM sizo-2.1(1573), sizo-2.2(1673), or control non-targeting siRNA (siNT) for 48 h (siZO-2.1 sense:5'-CCCAGAGACCAACAAGGAATT-3',antisense: 5'- UUCCUUGUUGGUCUCUGGGTT-3';siZO-2.2 sense：5'-GCAAUAUAUGGCCCUAAUATT-3',antisense: 5'-UAUUAGGGCCAUAUAUUGCTT-3';siNT：sense:5'-UUCUCCGAACGUGUCACGUTT-3',antisense: 5'-ACGUGACACGUUCGGAGAATT-3': were purchased from genepharma )by using Lipofectamine 2000 (Invitrogen)

**Western blot analysis**

Cells were lysed in lysis buffer with protease inhibitor cocktail (Roche Diagnostics) on ice for 30 min, centrifuged for 15 min at 12,000rpm. The supernatants were collected and protein concentrations were measured by the BCA method. Protein samples were run on a 10% polyacrylamide gel before transfer to a polyvinylidene difluoride membrane (Millipore, Life Science).Membranes were blocked with 5% milk for 1 h at room temperature and incubated with primary antibodies overnight and incubations of the membranes with primary antibodies and HRP-secondary antibody conjugates (Proteintech), the blots were developed by enhanced chemiluminescence (Millipore, Life Science). The antibodies were used as following: anti-ZO2 (1:1000; Cell Signaling Technology, Cat# #2847), anti-Actin (1:3000; Proteintech Group, Cat# 60008-1-Ig),anti-p53 (cell signaling technology, 1:1000) and anti-Actin (Proteintech Group, 1:3000),Goat Anti-rabbit IgG (H+L), HRP conjugated antibody (Proteintech Group, 1:3000), Goat Anti-mouse IgG (H+L).

**Immunofluorescence microscopy**

Cells were seeded onto coverslips and fixed with 4% paraformaldehyde for 30 min at room temperature, followed by permeabilization for 5 min with 0.1% Triton X-100 and blocked for 30 min with 10% FBS at room temperature. Cells were then incubated overnight with primary antibodies anti-F-actin (1:500; Cytoskeleton, Inc. Cat. # PHDG1-A) and anti-β-tubulin (1:300; Abcam, Cat. #ab195883) in 5% FBS at 4℃. After washing 3 times (10min each) with PBS, cells were analyzed using a Zeiss LSM 510 Meta laser scanning confocal microscope system.

**Cell counting kit-8 assay**

The proliferation of LO2 and HepG2 cells that were transfected with siNC or siTJP2 was analyzed using the cell counting kit-8 (CCK-8) (DOJINDO) according to the manufacture’s protocol. After 24h transfection, approximately 1 × 10^4^ cells were seeded in each well of 96-well plates. Next day, the [optical density](https://www.sciencedirect.com/topics/biochemistry-genetics-and-molecular-biology/optical-density) was detected at 0 h, 24 h, 48 h and 72 h after transfection by a Varioskan Flash instrument at 450 nm (BMG labtech Instruments).

**Cell death analysis**

Cell death of LO2 and HepG2 cells that were transfected with siNC or siTJP2 was analyzed by LIVE/DEAD Fixable Dead Cell Stain Kits (Life Technology) according to the manufacturer’s instructions. Centrifuge a Sample of cells in suspension containing at least 5 × 10^5^ cells, discard the supernatant. Then wash the cells once with PBS, and then incubated with LIVE/DEAD Fixable Dead Cell Stain in PBS for 30 min at room temperature in the dark. After washing with PBS with 1% FBS, cells were resuspended in PBS with 1% FBS and analyzed using flow cytometer (FACSAriaTM, BD).

# PART TWO. Tables

Supplement Table1. Twenty three pairs of TJP2 gene primers

| Gene | Exon | Primers | Sequence (5′-3′) | Fragment size(bp) |
| --- | --- | --- | --- | --- |
| TJP2 | 1 | 1F | AGCAGGAGCAGAAGCAGAAG | 221 |
|  |  | 1R | GGGAACAACTTCAGAGCAGC |  |
|  | 2 | 2F | TCATTGAAGGAGAGTGTGTTTGA | 291 |
|  |  | 2R | AAGATCCATGATCCGTAAAATCA |  |
|  | 3 | 3F | TGCTTGTAATAAATCCTGAAAGC | 250 |
|  |  | 3R | CCGTCTAGGGCCAACCAG |  |
|  | 4 | 4F | GCCACTAGACACTGAGCCCT | 300 |
|  |  | 4R | GGGTAATTTTCTTCTGGGCA |  |
|  | 5 | 5F | TTCCTGAAACCAGAACCAGG | 828 |
|  |  | 5R | AGATCACGCCACTTCCCTC |  |
|  | 6 | 6F | AGAGTTCAGGCCAGTCATCT | 370 |
|  |  | 6R | GCCACCTCTGTCTTACCACT |  |
|  | 7 | 7F | ATCCAGGCATGCAGGATTAT | 399 |
|  |  | 7R | AGAAGAATGGGGAAGCAACA |  |
|  | 8 | 8F | TGCAATTTCTCTGGGTAGGAG | 298 |
|  |  | 8R | TCCTCTCTGGAACTGAATCACA |  |
|  | 9 | 9F | GAGAAGCTGAAGGAAAGGCC | 337 |
|  |  | 9R | ACTCTTTGTTATGGCTGTGTGAG |  |
|  | 10 | 10F | TTTTGTGGATTTTGTGATTTTTCT | 238 |
|  |  | 10R | GGCAAAAACCACCACTCTTC |  |
|  | 11 | 11F | ACTCATCTCATGGTCCCAAGA | 483 |
|  |  | 11R | CAGAGACTTCAATGCTGGGC |  |
|  | 12 | 12F | ACAAAAGGGTCAGTGGCATC | 295 |
|  |  | 12R | TGGTACCAAGACACCTCTCTCA |  |
|  | 13 | 13F | AGGAGAAGCTGTGTTGAGTGTCT | 389 |
|  |  | 13R | TCATCATGTGGGGTTTCCTT |  |
|  | 14 | 14F | GGAAGTGAAGGTCCCCACAT | 677 |
|  |  | 14R | TGAACTTCTGAGCTCAAGTGATC |  |
|  | 15 | 15F | TGGTGAGGATAGTGAAGGCA | 369 |
|  |  | 15R | TTCCAGGTGAAGGGTACGTG |  |
|  | 16 | 16F | TGGGAGTATTTGGTTGTCACTG | 216 |
|  |  | 16R | TCCTAAAAGTGACTGCAGCA |  |
|  | 17 | 17F | TAATTGCTTGAACCCGGGAG | 544 |
|  |  | 17R | TTCAAGGAGTCATGAACACAA |  |
|  | 18 | 18F | TGCAAACATCTTCTTGCGTC | 300 |
|  |  | 18R | TCACTGCCCTCTTGAGAAAAG |  |
|  | 19 | 19F | GGGAATTTTCTTGAGTCCCC | 362 |
|  |  | 19R | AACACACAGCTGTCCACGAG |  |
|  | 20 | 20F | GCAGAACTCCTCCAAAGCAG | 281 |
|  |  | 20R | AGTGTGCATGCTCCTCCCT |  |
|  | 21 | 21F | GAAACTGATCAGGAAATGGAGTG | 500 |
|  |  | 21R | GGCTGTTCGCTGTTATGTTG |  |
|  | 22 | 22F | AGGAAACCAGCAAGCAGAGT | 226 |
|  |  | 22R | TTCTTTCTTCAAGTCCCAGTCC |  |
|  | 23 | 23F | GCAGAATGTGGCTCAGAGGT | 397 |
|  |  | 23R | CCATGGTGCATTCTAACTGG |  |

Supplement Table2. The top 28 KEGG pathways that are significantly enriched for DEGs in LO2 cells with TJP2 knockdown.

| KEGG_ID | Pathway_Name | S_Gene_Number | B_Gene_Number | p_Value | RichFactor |
| --- | --- | --- | --- | --- | --- |
| hsa03010 | Ribosome | 115 | 136 | 3.53755E-40 | 0.84558824 |
| hsa05016 | Huntington's disease | 110 | 192 | 2.50374E-15 | 0.57291667 |
| hsa05010 | Alzheimer's disease | 97 | 168 | 1.07417E-13 | 0.57738095 |
| hsa00190 | Oxidative phosphorylation | 74 | 133 | 3.1671E-09 | 0.55639098 |
| hsa04932 | Non-alcoholic fatty liver disease (NAFLD) | 81 | 151 | 3.23377E-09 | 0.53642384 |
| hsa05012 | Parkinson's disease | 72 | 142 | 8.21867E-07 | 0.50704225 |
| hsa04144 | Endocytosis | 99 | 241 | 0.000449973 | 0.41078838 |
| hsa05131 | Shigellosis | 34 | 64 | 0.001799148 | 0.53125 |
| hsa05200 | Pathways in cancer | 145 | 393 | 0.002492276 | 0.36895674 |
| hsa04120 | Ubiquitin mediated proteolysis | 60 | 137 | 0.00263463 | 0.4379562 |
| hsa04071 | Sphingolipid signaling pathway | 53 | 120 | 0.00501526 | 0.44166667 |
| hsa04621 | NOD-like receptor signaling pathway | 28 | 56 | 0.018715459 | 0.5 |
| hsa05223 | Non-small cell lung cancer | 28 | 56 | 0.018715459 | 0.5 |
| hsa04919 | Thyroid hormone signaling pathway | 49 | 115 | 0.018715459 | 0.42608696 |
| hsa05205 | Proteoglycans in cancer | 77 | 200 | 0.02174883 | 0.385 |
| hsa05222 | Small cell lung cancer | 38 | 85 | 0.022294273 | 0.44705882 |
| hsa04810 | Regulation of actin cytoskeleton | 80 | 210 | 0.022294273 | 0.38095238 |
| hsa05166 | HTLV-I infection | 94 | 254 | 0.022294273 | 0.37007874 |
| hsa03018 | RNA degradation | 35 | 77 | 0.023254746 | 0.45454545 |
| hsa05220 | Chronic myeloid leukemia | 33 | 72 | 0.02496293 | 0.45833333 |
| hsa04141 | Protein processing in endoplasmic reticulum | 66 | 169 | 0.02496293 | 0.39053254 |
| hsa00240 | Pyrimidine metabolism | 43 | 101 | 0.025941438 | 0.42574257 |
| hsa05132 | Salmonella infection | 36 | 83 | 0.042419152 | 0.43373494 |
| hsa05211 | Renal cell carcinoma | 30 | 66 | 0.042524433 | 0.45454545 |
| hsa03050 | Proteasome | 22 | 44 | 0.042958867 | 0.5 |
| hsa05100 | Bacterial invasion of epithelial cells | 34 | 78 | 0.045183719 | 0.43589744 |
| hsa04350 | TGF-beta signaling pathway | 36 | 84 | 0.045547167 | 0.42857143 |

Supplement Table3. The top 31 KEGG pathways that are significantly enriched for DEGs in HepG2 cells with TJP2 knockdown.

| KEGG_ID | Pathway_Name | S_Gene_Number | B_Gene_Number | p_Value | RichFactor |
| --- | --- | --- | --- | --- | --- |
| hsa01100 | Metabolic pathways | 104 | 1433 | 3.6718E-19 | 0.07257502 |
| hsa04151 | PI3K-Akt signaling pathway | 34 | 354 | 1.4446E-08 | 0.0960452 |
| hsa04979 | Cholesterol metabolism | 13 | 50 | 2.5195E-07 | 0.26 |
| hsa05165 | Human papillomavirus infection | 30 | 330 | 2.9426E-07 | 0.09090909 |
| hsa04610 | Complement and coagulation cascades | 15 | 79 | 3.86E-07 | 0.18987342 |
| hsa04512 | ECM-receptor interaction | 14 | 86 | 5.6263E-06 | 0.1627907 |
| hsa04145 | Phagosome | 18 | 152 | 6.3863E-06 | 0.11842105 |
| hsa04976 | Bile secretion | 12 | 72 | 2.7018E-05 | 0.16666667 |
| hsa04010 | MAPK signaling pathway | 24 | 295 | 2.9229E-05 | 0.08135593 |
| hsa05200 | Pathways in cancer | 34 | 530 | 2.9229E-05 | 0.06415094 |
| hsa03320 | PPAR signaling pathway | 12 | 76 | 3.274E-05 | 0.15789474 |
| hsa04152 | AMPK signaling pathway | 14 | 120 | 0.00010321 | 0.11666667 |
| hsa04514 | Cell adhesion molecules (CAMs) | 15 | 146 | 0.00018043 | 0.10273973 |
| hsa04350 | TGF-beta signaling pathway | 12 | 94 | 0.0001865 | 0.12765957 |
| hsa04014 | Ras signaling pathway | 19 | 232 | 0.00021675 | 0.08189655 |
| hsa04510 | Focal adhesion | 16 | 199 | 0.00120402 | 0.08040201 |
| hsa04915 | Estrogen signaling pathway | 13 | 138 | 0.00124604 | 0.0942029 |
| hsa05418 | Fluid shear stress and atherosclerosis | 13 | 139 | 0.00125926 | 0.09352518 |
| hsa00360 | Phenylalanine metabolism | 5 | 17 | 0.00195576 | 0.29411765 |
| hsa04975 | Fat digestion and absorption | 7 | 41 | 0.00195576 | 0.17073171 |
| hsa04066 | HIF-1 signaling pathway | 11 | 109 | 0.00195576 | 0.10091743 |
| hsa04810 | Regulation of actin cytoskeleton | 16 | 214 | 0.00195576 | 0.07476636 |
| hsa01230 | Biosynthesis of amino acids | 9 | 75 | 0.00216846 | 0.12 |
| hsa04668 | TNF signaling pathway | 11 | 112 | 0.00216846 | 0.09821429 |
| hsa05166 | Human T-cell leukemia virus 1 infection | 16 | 219 | 0.00216846 | 0.07305936 |
| hsa00980 | Metabolism of xenobiotics by cytochrome P450 | 9 | 76 | 0.00221576 | 0.11842105 |
| hsa00100 | Steroid biosynthesis | 5 | 19 | 0.00243547 | 0.26315789 |
| hsa04640 | Hematopoietic cell lineage | 10 | 97 | 0.00260223 | 0.10309278 |
| hsa04064 | NF-kappa B signaling pathway | 10 | 100 | 0.00314637 | 0.1 |
| hsa05204 | Chemical carcinogenesis | 9 | 82 | 0.00315595 | 0.1097561 |

Supplementary table 4 Normal *TJP2* and c.2668-11A>G variant translated proteins

1.Normal,NP_004808.2 tight junction protein ZO-2 isoform 1 [Homo sapiens](1190aa)

MPVRGDRGFPPRRELSGWLRAPGMEELIWEQYTVTLQKDSKRGFGIAVSGGRDNPHFENGETSIVISDVLPGGPADGLLQENDRVVMVNGTPMEDVLHSFAVQQLRKSGKVAAIVVKRPRKVQVAALQASPPLDQDDRAFEVMDEFDGRSFRSGYSERSRLNSHGGRSRSWEDSPERGRPHERARSRERDLSRDRSRGRSLERGLDQDHARTRDRSRGRSLERGLDHDFGPSRDRDRDRSRGRSIDQDYERAYHRAYDPDYERAYSPEYRRGARHDARSRGPRSRSREHPHSRSPSPEPRGRPGPIGVLLMKSRANEEYGLRLGSQIFVKEMTRTGLATKDGNLHEGDIILKINGTVTENMSLTDARKLIEKSRGKLQLVVLRDSQQTLINIPSLNDSDSEIEDISEIESNRSFSPEERRHQYSDYDYHSSSEKLKERPSSREDTPSRLSRMGATPTPFKSTGDIAGTVVPETNKEPRYQEDPPAPQPKAAPRTFLRPSPEDEAIYGPNTKMVRFKKGDSVGLRLAGGNDVGIFVAGIQEGTSAEQEGLQEGDQILKVNTQDFRGLVREDAVLYLLEIPKGEMVTILAQSRADVYRDILACGRGDSFFIRSHFECEKETPQSLAFTRGEVFRVVDTLYDGKLGNWLAVRIGNELEKGLIPNKSRAEQMASVQNAQRDNAGDRADFWRMRGQRSGVKKNLRKSREDLTAVVSVSTKFPAYERVLLREAGFKRPVVLFGPIADIAMEKLANELPDWFQTAKTEPKDAGSEKSTGVVRLNTVRQIIEQDKHALLDVTPKAVDLLNYTQWFPIVIFFNPDSRQGVKTMRQRLNPTSNKSSRKLFDQANKLKKTCAHLFTATINLNSANDSWFGSLKDTIQHQQGEAVWVSEGK**MEGMDDDPEDRMSYLTAMGADYLSCDSRLISDFEDTDGEGGAYTDNELDEPAEEPLVSSITRSSEPVQHEESIRKPSPEPRAQMRRAASSDQLRDNSPPPAFKPEPPKAKTQNKEESYDFSKSYEYKSNPSAVAGNETPGASTKGYPPPVAAKPTFGRSILKPSTPIPPQEGEEVGESSEEQDNAPKSVLGKVKIFEKMDHKARLQRMQELQEAQNARIEIAQKHPDIYAVPIKTHKPDPGTPQHTSSRPPEPQKAPSRPYQDTRGSYGSDAEEEEYRQQLSEHSKRGYYGQSARYRDTEL**

2.TJP2 **c.2668-11A>G** variant translated protein(897aa)

MPVRGDRGFPPRRELSGWLRAPGMEELIWEQYTVTLQKDSKRGFGIAVSGGRDNPHFENGETSIVISDVLPGGPADGLLQENDRVVMVNGTPMEDVLHSFAVQQLRKSGKVAAIVVKRPRKVQVAALQASPPLDQDDRAFEVMDEFDGRSFRSGYSERSRLNSHGGRSRSWEDSPERGRPHERARSRERDLSRDRSRGRSLERGLDQDHARTRDRSRGRSLERGLDHDFGPSRDRDRDRSRGRSIDQDYERAYHRAYDPDYERAYSPEYRRGARHDARSRGPRSRSREHPHSRSPSPEPRGRPGPIGVLLMKSRANEEYGLRLGSQIFVKEMTRTGLATKDGNLHEGDIILKINGTVTENMSLTDARKLIEKSRGKLQLVVLRDSQQTLINIPSLNDSDSEIEDISEIESNRSFSPEERRHQYSDYDYHSSSEKLKERPSSREDTPSRLSRMGATPTPFKSTGDIAGTVVPETNKEPRYQEDPPAPQPKAAPRTFLRPSPEDEAIYGPNTKMVRFKKGDSVGLRLAGGNDVGIFVAGIQEGTSAEQEGLQEGDQILKVNTQDFRGLVREDAVLYLLEIPKGEMVTILAQSRADVYRDILACGRGDSFFIRSHFECEKETPQSLAFTRGEVFRVVDTLYDGKLGNWLAVRIGNELEKGLIPNKSRAEQMASVQNAQRDNAGDRADFWRMRGQRSGVKKNLRKSREDLTAVVSVSTKFPAYERVLLREAGFKRPVVLFGPIADIAMEKLANELPDWFQTAKTEPKDAGSEKSTGVVRLNTVRQIIEQDKHALLDVTPKAVDLLNYTQWFPIVIFFNPDSRQGVKTMRQRLNPTSNKSSRKLFDQANKLKKTCAHLFTATINLNSANDSWFGSLKDTIQHQQGEAVWVSEGK**DSSNGRDG**

**PART THREE. Figures**

**Supplementary Figure1.** Sanger sequencing results of the two novel likely pathogenic variants.


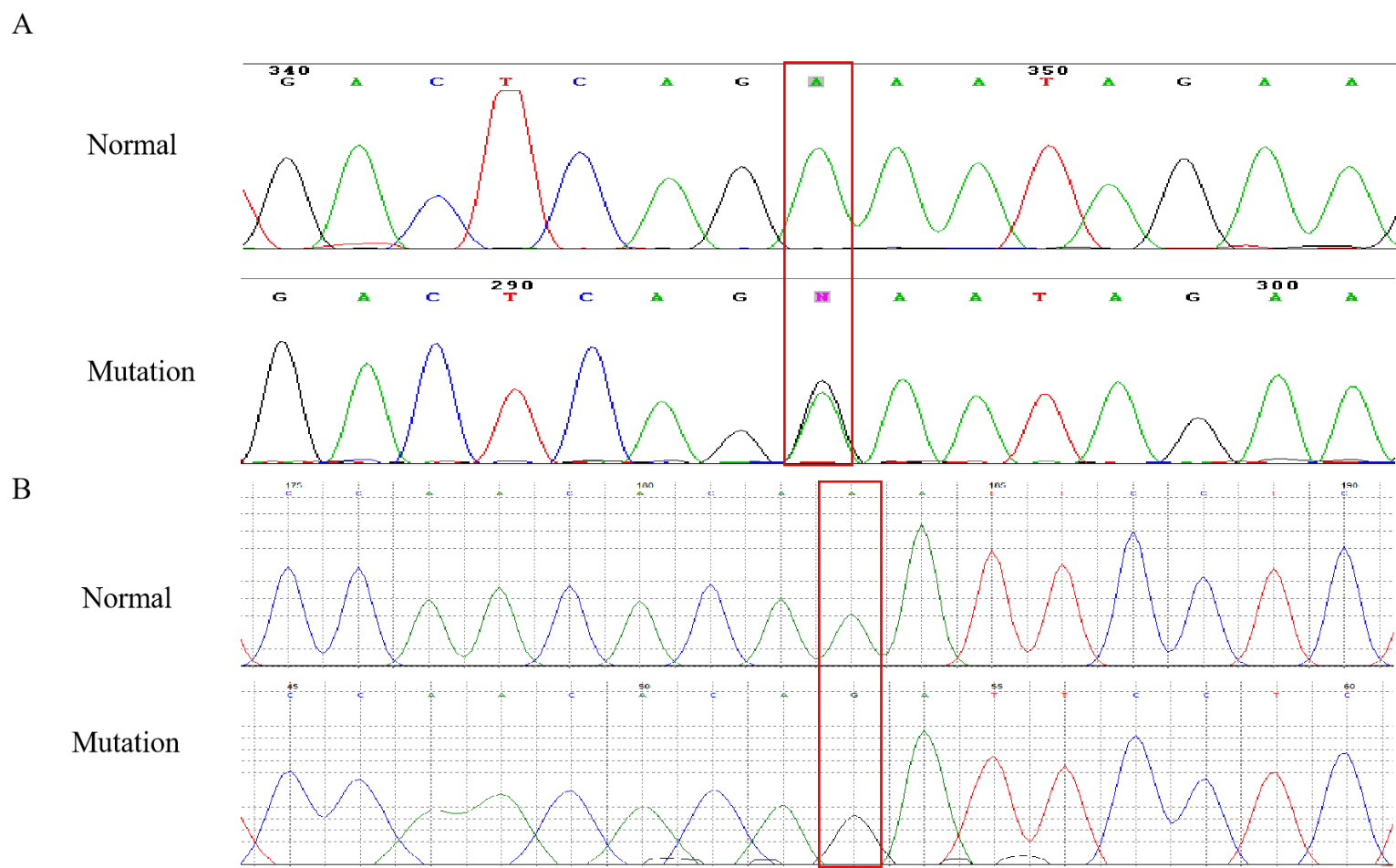


(A) Sanger sequencing confirmed wildtype (the upper panel) and heterozygous *TJP2* c.1202A>G variant (the lower panel) in PFIC patients . (B) Sanger sequencing confirmed wildtype (the upper panel) and homozygous *TJP2* c.2668-11A>G variant (the lower panel) in PFIC patients.

**Supplementary Figure2. The splicing effects of *TJP2* c.2668-11A>G predicted by Alamut Visual software.**


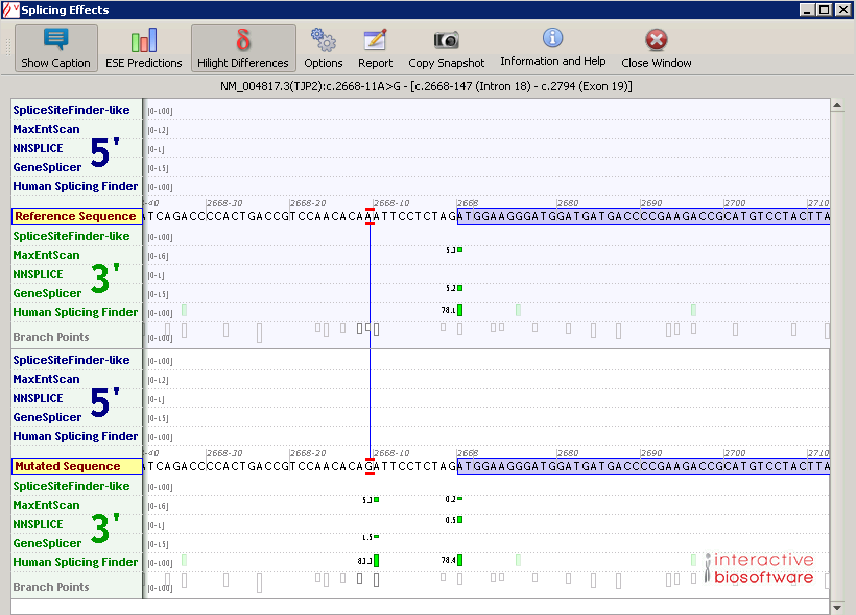


**Supplementary Figure3. Schematic of mini-gene system and agarose gel electrophoresis results of amplified products**


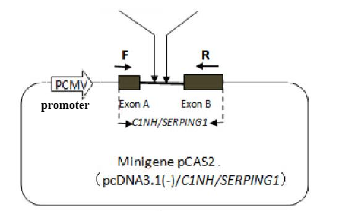

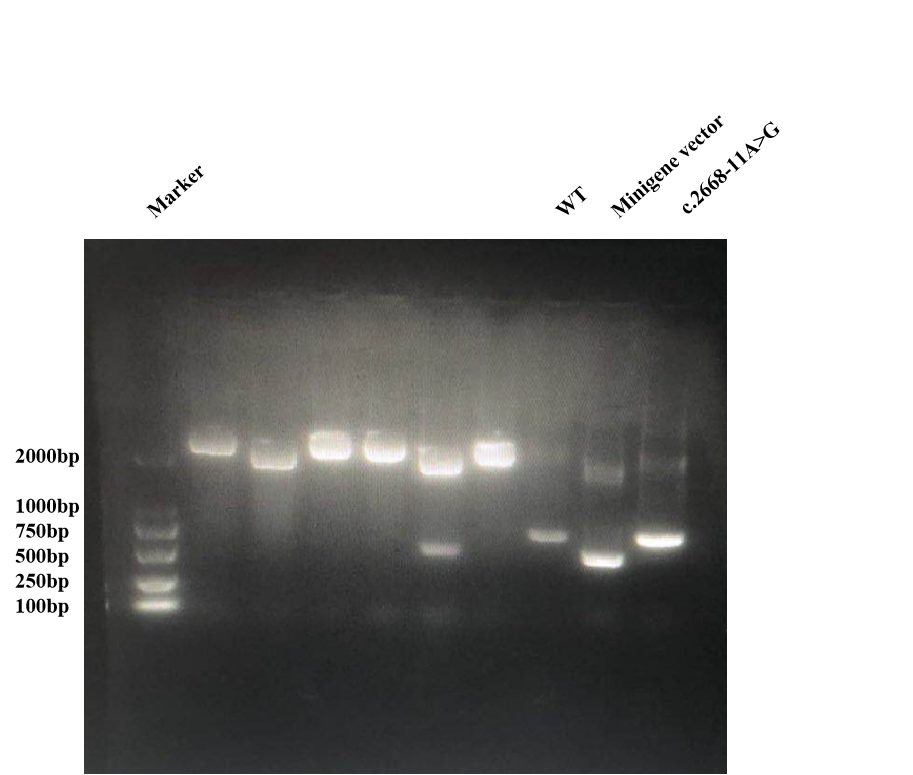


The RT-PCR product was used as a template, and the universal primer pCAS2-RT-F/R1 was used for PCR amplification. On 2% agarose gel electrophoresis, the Minigene vector was seen at the 474bp band and the wildtype at 687bp. The *TJP2* c.2668-11A>G variant of the band is 697bp.
