# Supplementary material for: Two Novel Pathogenic Variants of TJP2 Gene and the Underlying Molecular Mechanisms in Progressive Familial Intrahepatic Cholestasis Type 4 Patients
Source: Front Cell Dev Biol. 2021 Aug 24;9:661599. doi: 10.3389/fcell.2021.661599 (PMC8421653; doi:10.3389/fcell.2021.661599)
Supplement: Supplementary file 5 [file Table_1.doc]

Supplement Table1. Twenty three pairs of TJP2 gene primers

| Gene | Exon | Primers | Sequence (5′-3′) | Fragment size(bp) |
| --- | --- | --- | --- | --- |
| TJP2 | 1 | 1F | AGCAGGAGCAGAAGCAGAAG | 221 |
|  |  | 1R | GGGAACAACTTCAGAGCAGC |  |
|  | 2 | 2F | TCATTGAAGGAGAGTGTGTTTGA | 291 |
|  |  | 2R | AAGATCCATGATCCGTAAAATCA |  |
|  | 3 | 3F | TGCTTGTAATAAATCCTGAAAGC | 250 |
|  |  | 3R | CCGTCTAGGGCCAACCAG |  |
|  | 4 | 4F | GCCACTAGACACTGAGCCCT | 300 |
|  |  | 4R | GGGTAATTTTCTTCTGGGCA |  |
|  | 5 | 5F | TTCCTGAAACCAGAACCAGG | 828 |
|  |  | 5R | AGATCACGCCACTTCCCTC |  |
|  | 6 | 6F | AGAGTTCAGGCCAGTCATCT | 370 |
|  |  | 6R | GCCACCTCTGTCTTACCACT |  |
|  | 7 | 7F | ATCCAGGCATGCAGGATTAT | 399 |
|  |  | 7R | AGAAGAATGGGGAAGCAACA |  |
|  | 8 | 8F | TGCAATTTCTCTGGGTAGGAG | 298 |
|  |  | 8R | TCCTCTCTGGAACTGAATCACA |  |
|  | 9 | 9F | GAGAAGCTGAAGGAAAGGCC | 337 |
|  |  | 9R | ACTCTTTGTTATGGCTGTGTGAG |  |
|  | 10 | 10F | TTTTGTGGATTTTGTGATTTTTCT | 238 |
|  |  | 10R | GGCAAAAACCACCACTCTTC |  |
|  | 11 | 11F | ACTCATCTCATGGTCCCAAGA | 483 |
|  |  | 11R | CAGAGACTTCAATGCTGGGC |  |
|  | 12 | 12F | ACAAAAGGGTCAGTGGCATC | 295 |
|  |  | 12R | TGGTACCAAGACACCTCTCTCA |  |
|  | 13 | 13F | AGGAGAAGCTGTGTTGAGTGTCT | 389 |
|  |  | 13R | TCATCATGTGGGGTTTCCTT |  |
|  | 14 | 14F | GGAAGTGAAGGTCCCCACAT | 677 |
|  |  | 14R | TGAACTTCTGAGCTCAAGTGATC |  |
|  | 15 | 15F | TGGTGAGGATAGTGAAGGCA | 369 |
|  |  | 15R | TTCCAGGTGAAGGGTACGTG |  |
|  | 16 | 16F | TGGGAGTATTTGGTTGTCACTG | 216 |
|  |  | 16R | TCCTAAAAGTGACTGCAGCA |  |
|  | 17 | 17F | TAATTGCTTGAACCCGGGAG | 544 |
|  |  | 17R | TTCAAGGAGTCATGAACACAA |  |
|  | 18 | 18F | TGCAAACATCTTCTTGCGTC | 300 |
|  |  | 18R | TCACTGCCCTCTTGAGAAAAG |  |
|  | 19 | 19F | GGGAATTTTCTTGAGTCCCC | 362 |
|  |  | 19R | AACACACAGCTGTCCACGAG |  |
|  | 20 | 20F | GCAGAACTCCTCCAAAGCAG | 281 |
|  |  | 20R | AGTGTGCATGCTCCTCCCT |  |
|  | 21 | 21F | GAAACTGATCAGGAAATGGAGTG | 500 |
|  |  | 21R | GGCTGTTCGCTGTTATGTTG |  |
|  | 22 | 22F | AGGAAACCAGCAAGCAGAGT | 226 |
|  |  | 22R | TTCTTTCTTCAAGTCCCAGTCC |  |
|  | 23 | 23F | GCAGAATGTGGCTCAGAGGT | 397 |
|  |  | 23R | CCATGGTGCATTCTAACTGG |  |
