# Supplementary material for: Two Novel Pathogenic Variants of TJP2 Gene and the Underlying Molecular Mechanisms in Progressive Familial Intrahepatic Cholestasis Type 4 Patients
Source: Front Cell Dev Biol. 2021 Aug 24;9:661599. doi: 10.3389/fcell.2021.661599 (PMC8421653; doi:10.3389/fcell.2021.661599)
Supplement: Supplementary file 6 [file Table_2.doc]

Supplement Table2. The top 28 KEGG pathways that are significantly enriched for DEGs in LO2 cells with TJP2 knockdown.

| KEGG_ID | Pathway_Name | S_Gene_Number | B_Gene_Number | p_Value | RichFactor |
| --- | --- | --- | --- | --- | --- |
| hsa03010 | Ribosome | 115 | 136 | 3.53755E-40 | 0.84558824 |
| hsa05016 | Huntington's disease | 110 | 192 | 2.50374E-15 | 0.57291667 |
| hsa05010 | Alzheimer's disease | 97 | 168 | 1.07417E-13 | 0.57738095 |
| hsa00190 | Oxidative phosphorylation | 74 | 133 | 3.1671E-09 | 0.55639098 |
| hsa04932 | Non-alcoholic fatty liver disease (NAFLD) | 81 | 151 | 3.23377E-09 | 0.53642384 |
| hsa05012 | Parkinson's disease | 72 | 142 | 8.21867E-07 | 0.50704225 |
| hsa04144 | Endocytosis | 99 | 241 | 0.000449973 | 0.41078838 |
| hsa05131 | Shigellosis | 34 | 64 | 0.001799148 | 0.53125 |
| hsa05200 | Pathways in cancer | 145 | 393 | 0.002492276 | 0.36895674 |
| hsa04120 | Ubiquitin mediated proteolysis | 60 | 137 | 0.00263463 | 0.4379562 |
| hsa04071 | Sphingolipid signaling pathway | 53 | 120 | 0.00501526 | 0.44166667 |
| hsa04621 | NOD-like receptor signaling pathway | 28 | 56 | 0.018715459 | 0.5 |
| hsa05223 | Non-small cell lung cancer | 28 | 56 | 0.018715459 | 0.5 |
| hsa04919 | Thyroid hormone signaling pathway | 49 | 115 | 0.018715459 | 0.42608696 |
| hsa05205 | Proteoglycans in cancer | 77 | 200 | 0.02174883 | 0.385 |
| hsa05222 | Small cell lung cancer | 38 | 85 | 0.022294273 | 0.44705882 |
| hsa04810 | Regulation of actin cytoskeleton | 80 | 210 | 0.022294273 | 0.38095238 |
| hsa05166 | HTLV-I infection | 94 | 254 | 0.022294273 | 0.37007874 |
| hsa03018 | RNA degradation | 35 | 77 | 0.023254746 | 0.45454545 |
| hsa05220 | Chronic myeloid leukemia | 33 | 72 | 0.02496293 | 0.45833333 |
| hsa04141 | Protein processing in endoplasmic reticulum | 66 | 169 | 0.02496293 | 0.39053254 |
| hsa00240 | Pyrimidine metabolism | 43 | 101 | 0.025941438 | 0.42574257 |
| hsa05132 | Salmonella infection | 36 | 83 | 0.042419152 | 0.43373494 |
| hsa05211 | Renal cell carcinoma | 30 | 66 | 0.042524433 | 0.45454545 |
| hsa03050 | Proteasome | 22 | 44 | 0.042958867 | 0.5 |
| hsa05100 | Bacterial invasion of epithelial cells | 34 | 78 | 0.045183719 | 0.43589744 |
| hsa04350 | TGF-beta signaling pathway | 36 | 84 | 0.045547167 | 0.42857143 |
