# Supplementary material for: Two Novel Pathogenic Variants of TJP2 Gene and the Underlying Molecular Mechanisms in Progressive Familial Intrahepatic Cholestasis Type 4 Patients
Source: Front Cell Dev Biol. 2021 Aug 24;9:661599. doi: 10.3389/fcell.2021.661599 (PMC8421653; doi:10.3389/fcell.2021.661599)
Supplement: Supplementary file 7 [file Table_3.doc]

Supplement Table3. The top 31 KEGG pathways that are significantly enriched for DEGs in HepG2 cells with TJP2 knockdown.

| KEGG_ID | Pathway_Name | S_Gene_Number | B_Gene_Number | p_Value | RichFactor |
| --- | --- | --- | --- | --- | --- |
| hsa01100 | Metabolic pathways | 104 | 1433 | 3.6718E-19 | 0.07257502 |
| hsa04151 | PI3K-Akt signaling pathway | 34 | 354 | 1.4446E-08 | 0.0960452 |
| hsa04979 | Cholesterol metabolism | 13 | 50 | 2.5195E-07 | 0.26 |
| hsa05165 | Human papillomavirus infection | 30 | 330 | 2.9426E-07 | 0.09090909 |
| hsa04610 | Complement and coagulation cascades | 15 | 79 | 3.86E-07 | 0.18987342 |
| hsa04512 | ECM-receptor interaction | 14 | 86 | 5.6263E-06 | 0.1627907 |
| hsa04145 | Phagosome | 18 | 152 | 6.3863E-06 | 0.11842105 |
| hsa04976 | Bile secretion | 12 | 72 | 2.7018E-05 | 0.16666667 |
| hsa04010 | MAPK signaling pathway | 24 | 295 | 2.9229E-05 | 0.08135593 |
| hsa05200 | Pathways in cancer | 34 | 530 | 2.9229E-05 | 0.06415094 |
| hsa03320 | PPAR signaling pathway | 12 | 76 | 3.274E-05 | 0.15789474 |
| hsa04152 | AMPK signaling pathway | 14 | 120 | 0.00010321 | 0.11666667 |
| hsa04514 | Cell adhesion molecules (CAMs) | 15 | 146 | 0.00018043 | 0.10273973 |
| hsa04350 | TGF-beta signaling pathway | 12 | 94 | 0.0001865 | 0.12765957 |
| hsa04014 | Ras signaling pathway | 19 | 232 | 0.00021675 | 0.08189655 |
| hsa04510 | Focal adhesion | 16 | 199 | 0.00120402 | 0.08040201 |
| hsa04915 | Estrogen signaling pathway | 13 | 138 | 0.00124604 | 0.0942029 |
| hsa05418 | Fluid shear stress and atherosclerosis | 13 | 139 | 0.00125926 | 0.09352518 |
| hsa00360 | Phenylalanine metabolism | 5 | 17 | 0.00195576 | 0.29411765 |
| hsa04975 | Fat digestion and absorption | 7 | 41 | 0.00195576 | 0.17073171 |
| hsa04066 | HIF-1 signaling pathway | 11 | 109 | 0.00195576 | 0.10091743 |
| hsa04810 | Regulation of actin cytoskeleton | 16 | 214 | 0.00195576 | 0.07476636 |
| hsa01230 | Biosynthesis of amino acids | 9 | 75 | 0.00216846 | 0.12 |
| hsa04668 | TNF signaling pathway | 11 | 112 | 0.00216846 | 0.09821429 |
| hsa05166 | Human T-cell leukemia virus 1 infection | 16 | 219 | 0.00216846 | 0.07305936 |
| hsa00980 | Metabolism of xenobiotics by cytochrome P450 | 9 | 76 | 0.00221576 | 0.11842105 |
| hsa00100 | Steroid biosynthesis | 5 | 19 | 0.00243547 | 0.26315789 |
| hsa04640 | Hematopoietic cell lineage | 10 | 97 | 0.00260223 | 0.10309278 |
| hsa04064 | NF-kappa B signaling pathway | 10 | 100 | 0.00314637 | 0.1 |
| hsa05204 | Chemical carcinogenesis | 9 | 82 | 0.00315595 | 0.1097561 |
