# Supplementary material for: Two Novel Pathogenic Variants of TJP2 Gene and the Underlying Molecular Mechanisms in Progressive Familial Intrahepatic Cholestasis Type 4 Patients
Source: Front Cell Dev Biol. 2021 Aug 24;9:661599. doi: 10.3389/fcell.2021.661599 (PMC8421653; doi:10.3389/fcell.2021.661599)
Supplement: Supplementary file 8 [file Table_4.doc]

Supplementary table 4. Normal *TJP2* and c.2668-11A>G variant translated proteins

1.Normal,NP_004808.2 tight junction protein ZO-2 isoform 1 [Homo sapiens](1190aa)

MPVRGDRGFPPRRELSGWLRAPGMEELIWEQYTVTLQKDSKRGFGIAVSGGRDNPHFENGETSIVISDVLPGGPADGLLQENDRVVMVNGTPMEDVLHSFAVQQLRKSGKVAAIVVKRPRKVQVAALQASPPLDQDDRAFEVMDEFDGRSFRSGYSERSRLNSHGGRSRSWEDSPERGRPHERARSRERDLSRDRSRGRSLERGLDQDHARTRDRSRGRSLERGLDHDFGPSRDRDRDRSRGRSIDQDYERAYHRAYDPDYERAYSPEYRRGARHDARSRGPRSRSREHPHSRSPSPEPRGRPGPIGVLLMKSRANEEYGLRLGSQIFVKEMTRTGLATKDGNLHEGDIILKINGTVTENMSLTDARKLIEKSRGKLQLVVLRDSQQTLINIPSLNDSDSEIEDISEIESNRSFSPEERRHQYSDYDYHSSSEKLKERPSSREDTPSRLSRMGATPTPFKSTGDIAGTVVPETNKEPRYQEDPPAPQPKAAPRTFLRPSPEDEAIYGPNTKMVRFKKGDSVGLRLAGGNDVGIFVAGIQEGTSAEQEGLQEGDQILKVNTQDFRGLVREDAVLYLLEIPKGEMVTILAQSRADVYRDILACGRGDSFFIRSHFECEKETPQSLAFTRGEVFRVVDTLYDGKLGNWLAVRIGNELEKGLIPNKSRAEQMASVQNAQRDNAGDRADFWRMRGQRSGVKKNLRKSREDLTAVVSVSTKFPAYERVLLREAGFKRPVVLFGPIADIAMEKLANELPDWFQTAKTEPKDAGSEKSTGVVRLNTVRQIIEQDKHALLDVTPKAVDLLNYTQWFPIVIFFNPDSRQGVKTMRQRLNPTSNKSSRKLFDQANKLKKTCAHLFTATINLNSANDSWFGSLKDTIQHQQGEAVWVSEGK**MEGMDDDPEDRMSYLTAMGADYLSCDSRLISDFEDTDGEGGAYTDNELDEPAEEPLVSSITRSSEPVQHEESIRKPSPEPRAQMRRAASSDQLRDNSPPPAFKPEPPKAKTQNKEESYDFSKSYEYKSNPSAVAGNETPGASTKGYPPPVAAKPTFGRSILKPSTPIPPQEGEEVGESSEEQDNAPKSVLGKVKIFEKMDHKARLQRMQELQEAQNARIEIAQKHPDIYAVPIKTHKPDPGTPQHTSSRPPEPQKAPSRPYQDTRGSYGSDAEEEEYRQQLSEHSKRGYYGQSARYRDTEL**

2.TJP2 **c.2668-11A>G** variant translated protein(897aa)

MPVRGDRGFPPRRELSGWLRAPGMEELIWEQYTVTLQKDSKRGFGIAVSGGRDNPHFENGETSIVISDVLPGGPADGLLQENDRVVMVNGTPMEDVLHSFAVQQLRKSGKVAAIVVKRPRKVQVAALQASPPLDQDDRAFEVMDEFDGRSFRSGYSERSRLNSHGGRSRSWEDSPERGRPHERARSRERDLSRDRSRGRSLERGLDQDHARTRDRSRGRSLERGLDHDFGPSRDRDRDRSRGRSIDQDYERAYHRAYDPDYERAYSPEYRRGARHDARSRGPRSRSREHPHSRSPSPEPRGRPGPIGVLLMKSRANEEYGLRLGSQIFVKEMTRTGLATKDGNLHEGDIILKINGTVTENMSLTDARKLIEKSRGKLQLVVLRDSQQTLINIPSLNDSDSEIEDISEIESNRSFSPEERRHQYSDYDYHSSSEKLKERPSSREDTPSRLSRMGATPTPFKSTGDIAGTVVPETNKEPRYQEDPPAPQPKAAPRTFLRPSPEDEAIYGPNTKMVRFKKGDSVGLRLAGGNDVGIFVAGIQEGTSAEQEGLQEGDQILKVNTQDFRGLVREDAVLYLLEIPKGEMVTILAQSRADVYRDILACGRGDSFFIRSHFECEKETPQSLAFTRGEVFRVVDTLYDGKLGNWLAVRIGNELEKGLIPNKSRAEQMASVQNAQRDNAGDRADFWRMRGQRSGVKKNLRKSREDLTAVVSVSTKFPAYERVLLREAGFKRPVVLFGPIADIAMEKLANELPDWFQTAKTEPKDAGSEKSTGVVRLNTVRQIIEQDKHALLDVTPKAVDLLNYTQWFPIVIFFNPDSRQGVKTMRQRLNPTSNKSSRKLFDQANKLKKTCAHLFTATINLNSANDSWFGSLKDTIQHQQGEAVWVSEGK**DSSNGRDG**
